# Clinical utility of measuring expression levels of *KAP1*, *TIMP1* and *STC2* in peripheral blood of patients with gastric cancer

**DOI:** 10.1186/1477-7819-11-81

**Published:** 2013-04-02

**Authors:** Yuan-Yu Wang, Li Li, Zhong-Sheng Zhao, Hui-Ju Wang

**Affiliations:** 1 Department of Gastrointestinal Surgery, Zhejiang Provincial People’s Hospital, 158 shangtang road, Hangzhou, Zhejiang, 310014, PR China; 2 Department of Pathology, Zhejiang Provincial People’s Hospital, 158 shangtang road, Hangzhou, Zhejiang, 310014, PR China; 3 Key Laboratory of Gastroenterology of Zhejiang Province, 158 shangtang road, Hangzhou, Zhejiang, 310014, PR China

**Keywords:** Gastric cancer, Kinesin II-associated protein, TIMP metallopeptidase inhibitor 1, Stanniocalcin 2

## Abstract

**Background:**

We examined preoperative kinesin II-associated protein (*KAP1*), TIMP metallopeptidase inhibitor 1 (*TIMP1*) and stanniocalcin 2 (*STC2*) expression levels in patients with gastric cancers to assess their clinical application for diagnosing and monitoring diseases.

**Methods:**

Real-time reverse transcription-polymerase chain reaction was used to detect the expression levels of *KAP1*, *TIMP1*, *STC2*, talin 2 (*TLN2*), sushi-repeat-containing protein, X-linked 2 (*SRPX2*) and secreted protein, acidic, cysteine-rich (*SPARC*) in the patients’ peripheral blood karyocytes. The data were analyzed with receiver operating characteristics (ROC) curves.

**Results:**

A total of 112 patients with gastric cancer, 42 patients with recurrence and 107 healthy volunteers were recruited. There were significant correlations between *KAP1*, *TIMP1* and *STC2* levels, and TNM tumor stages and distant metastases. The area under the ROC curves (AUC) of *KAP1* was 0.803 ± 0.040 (*P* = 0.0001), the AUC of *TIMP1* was 0.767 ± 0.043 (*P* = 0.0001) and the AUC of *STC2* was 0.769 ± 0.045 (*P* = 0.0001), thus differentiating preoperative gastric cancer patients from healthy volunteers by ROC curve analysis. The AUC of *STC2* was 0.739 ± 0.070 (*P* = 0.004) and the AUC of *KAP1* was 0.418 ± 0.088 (*P* = 0.319), thus differentiating recurrence of gastric cancer from healthy volunteers by ROC curve analysis. High *TIMP1* and *STC2* expression levels were suspected to be poor prognostic factors of disease recurrence in patients with gastric cancer.

**Conclusions:**

*KAP1*, *TIMP1* and *STC2* expression levels may be potential biomarkers for the screening, diagnosis, prognosis and surveillance of gastric cancer.

## Background

The overall 5-year survival rate in China for patients with gastric cancer is low, at 40%, and the rate of lymph node metastasis is higher (50 to 75%) [1]. Patients who had undergone potentially curative surgery retain the risk of recurrence mainly because of tumor dissemination via the blood or lymphatic circulations. To improve the cure rates for patients with gastric cancer, the primary tumors must be detected at an early stage, and recurrent disease must be diagnosed while it is still minimal or clinically occult; micrometastases are currently undetectable by conventional methods [2,3]. Conventional serum markers, however, lack sufficient sensitivity and specificity to facilitate early detection of cancer. To improve the poor survival outcome and to permit earlier diagnosis, there is a need for new and more sensitive biomarkers than those currently available, such as carcinoembryonic antigen and CA19-9 [4].

TIMP metallopeptidase inhibitor 1 (*TIMP1*) belongs to the tissue inhibitor of metalloproteases (*TIMP*) gene family. The proteins encoded by this gene family are natural inhibitors of the matrix metalloproteinases (MMPs), a group of peptidases involved in degradation of the extracellular matrix. In addition to its inhibitory role against most of the known MMPs, the encoded protein is also able to promote cell proliferation in a wide range of cell types, and may also have an anti-apoptotic function. *TIMP1* has been shown to be overexpressed in both liver and peritoneal metastases from patients with colorectal adenocarcinoma [5] and malignant thyroid neoplasms [6].

Stanniocalcin 2 (*STC2*) encodes a secreted, homodimeric glycoprotein that is expressed in a wide variety of tissues and may have autocrine or paracrine functions. The protein may play a role in the regulation of renal and intestinal calcium and phosphate transport, cell metabolism, or cellular calcium/phosphate homeostasis. Constitutive overexpression of human *STC2* in mice resulted in pre- and postnatal growth restriction, reduced bone and skeletal muscle growth, and organomegaly. Expression of this gene is induced by estrogen and altered in some breast cancers [7]. The expression of *STC2* was significantly correlated with lymph node metastasis, lymphatic invasion, and distant metastasis of esophageal squamous cell cancer [8] and colorectal cancer [9].

The protein encoded by *KAP1* mediates transcriptional control by interacting with the Kruppel-associated box repression domain found in many transcription factors. The protein, a member of the tripartite motif family, localizes to the nucleus and is thought to associate with specific chromatin regions. This tripartite motif includes three zinc-binding domains, a RING, a B-box type 1 and a B-box type 2, and a coiled-coil region. KAP1 plays an important role in progression to peritoneal carcinomatosis in gastric cancer patients [10].

In our previously study, *KAP1*, *TIMP1*, *STC2*, talin 2 (*TLN2*), sushi-repeat-containing protein, X-linked 2 (*SRPX2*), *ITGB1* and secreted protein, acidic, cysteine-rich (*SPARC*) were selected by the Affymetrix GeneChip™ HG-U133A2.0 array, and were upregulated (ratio ≥2) in gastric tumor tissue [11] (Affymetrix, Santa Clara, California, United States). In this study, we detected the expression of *KAP1*, *TIMP1*, *STC2*, *TLN2*, *SRPX2*, integrin beta 1 (*ITGB1*) and *SPARC* mRNAs in peripheral blood samples from pre-operative gastric cancer patients, patients with recurrence, and healthy volunteers. We compared the relationships between these results and clinical findings to assess the diagnostic value of the biomarkers in these patients.

## Methods

### Patients and peripheral blood samples

A total of 112 patients with gastric cancer and 42 patients with recurrence, diagnosed and treated between January 2006 and December 2010 at Zhejiang Provincial People’s Hospital, were enrolled in this study. The median age of the gastric cancer patients was 60 years (range, 36 to 87 years), and the study comprised 77 males and 35 females. The median age of patients with recurrence was 64 years (range, 51 to 65 years), and this subset comprised 31 males and 11 females. One hundred and seven healthy volunteers who visited the hospital for a health examination and who had normal appearance of the gastric mucosa on gastroscopic examination were also enrolled. The healthy controls comprised 76 males and 31 females, with a median age of 59 years (range, 35 to 86 years).

All patients had follow-up records from the date of their operation, with a follow-up deadline of November 2011. None of the 112 patients with gastric cancer had received preoperative chemotherapy or radiotherapy, but were receiving radical or palliative gastrectomy. Seventy of these patients (62.5%) were resected with tumor-free resection margins (R0), while the remaining 42 patients (37.5%) were resected with R1 or R2. Following surgery, patients with stage I to III tumors received adjuvant chemotherapy, while patients with stage IV tumors received systemic chemotherapy with various combinations of regimens. Ten patients with recurrence received a radical operation again, and all received systemic chemotherapy. Of the 112 gastric patients with cancer, 12.5% (14 of 112) were classified as stage I, 35.71% (40 of 112) were stage II, 34.82% (39 of 112) were stage III, and 16.96% (19 of 112) were stage IV (Table 1). The clinicopathological findings were determined according to classification of malignant tumors as set out by the World Health Organization for gastric cancer. The study was approved and monitored by the ethics committee of our hospitals, and informed consent was obtained from each patient and healthy volunteer.

**Table 1:** The characterization of the patient groups

	**Healthy volunteers**	**Gastric cancer**	**Recurrent patients**
Age (range)	59 y (35 to 86 y)	60 y (36 to 87 y)	64 y (51 to 65 y)
Gender			
Female	31	35	11
Male	76	77	31
TNM Stage			
I		14	0
II		40	13
III		39	26
IV		19	3
Resection margins			
R0		70	42
R1		17	
R2		23	

### Total RNA preparation

A 2-ml sample of peripheral blood from all of subjects was collected in test tubes containing sodium citrate. Total RNA from peripheral blood karyocyte was isolated using QIAamp RNA Blood Mini Kits (Cat. No: 52304, QIAGEN, Hilden, Germany) following the manufacturer’s protocol.

### Real-time quantitative PCR

The expressions of *KAP1*, *TIMP1*, *STC2*, *TLN2*, *SRPX2* and *SPARC* in peripheral blood from 82 patients with gastric cancer, 24 patients with recurrence and 69 healthy volunteers were confirmed by RT-PCR(reverse transcription PCR). Total RNA was extracted and cDNAs were reverse-transcribed by RevertAid™ reverse transcriptase. Real-time PCR was carried out using the ABI PRISM 7700 Sequence Detection System (Life Technologies, Grand Island, New York, United States) at 50°C for 2 min, 95°C for 10 min, followed by 50 cycles at 95°C for 15 s, and 60°C for 1 min. The primers are listed in Table 2. The expression of *GAPDH* was used to normalize that of the target genes. Each assay was done in triplicate, the average was calculated, and the expression level of targets mRNA was expressed as 2^–ΔCt^, *ΔCt* = *Ct*(*targets mRNA*) − *Ct*(*GAPDH*).

**Table 2:** Primers used in real-time quantitative PCR

**Gene**	**F**	**R**	**Lengths**
*KAP1*	GAAGGCTATGGCTTTGGGTC	CAGGCGTTCAAGGCTCACT	165 bp
*TIMP1*	CCTGTTGTTGCTGTGGCTGAT	GGTTGTGGGACCTGTGGAAGTA	272 bp
*STC2*	CATCCTTATCCGTCAACTCATCAG	TACGCTTGGTTTCTTGGTGTCT	71 bp
*TALIN1*	TGAAGCGAGCCTCAGATAAT	CACCACCACTGTCTCATTCTC	83 bp
*SRPX2*	CGCTGCCCAACTCTGAAACCTC	ACCACTGGCGGCTGGACTGA	151 bp
*SPARC*	TTCTCACATAAGCCCAGTTCA	GAACAACCGATTCACCAACTC	406 bp
*GAPDH*	TGAAGGTCGGAGTCAACGG	CTGGAAGATGGTGATGGGATT	195 bp

### Statistical analysis

Statistical analysis was performed using SPSS 16.0 (SPSS Inc, Chicago, United States). The level of significance was set at *P* < 0.05. ROC (receiver operation characteristics) curves were constructed by calculating the sensitivities and specificities of a biomarker or the diagnostic score of a logistical regression model at different cut-off points for differentiating gastric cancer cases from healthy volunteers. The area under the ROC curves (AUC) was statistically interpreted as the probability to correctly distinguish patients with gastric cancer from normal subjects. An area of 1.0 represented a perfect test, and an area of 0.5 represented a worthless test. The *P* value was the probability that the sample AUC was found when, in fact, the true (population) AUC was 0.5 (null hypothesis: area = 0.5). If *P* was low (*P* < 0.05), then it was concluded that the AUC was significantly different between the two groups. The ROC curves were used to determine the optimal cut-off values (with the Youden J test for overall accuracy) [12]. The cutoff value corresponded to the optimal diagnostic accuracy (the highest sum of the respective values for sensitivity and specificity) [6].

## Results

### The diagnostic efficacy of *KAP1*, *TIMP1*, *STC2*, *TLN2*, *SRPX2* and *SPARC* in patients with gastric cancer

**Figure 1:**
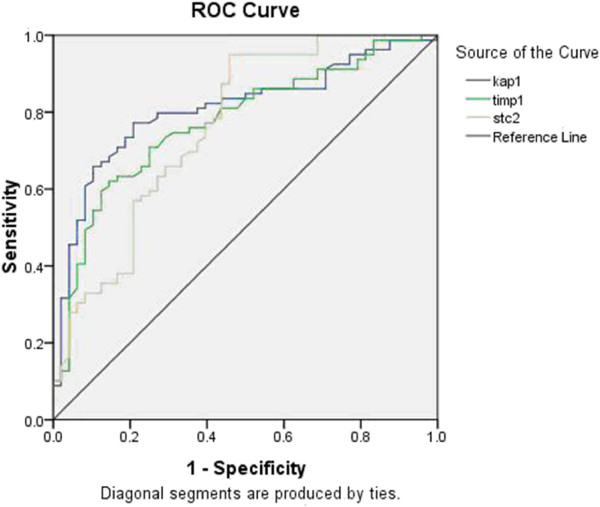
**Receiver operation characteristics (ROC) curve of *KAP1*, *TIMP1 *and *STC2 *in peripheral blood of patients with gastric cancer and healthy volunteers.** The clinical values were assessed by differentiating 112 preoperative gastric cancer patients from 107 healthy volunteers.

To evaluate the diagnostic value of *KAP1*, *TIMP1*, *STC2*, *TLN2*, *SRPX2*, *ITGB1* and *SPARC* expression levels in the diagnosis of patients with gastric cancer, the AUC value from ROC curve analysis was determined (Figure 1). The clinical values were assessed by differentiating preoperative gastric cancer patients from healthy volunteers. The AUC of *KAP1* was 0.803 ± 0.040 (*P* = 0.0001; 95% CI 0.724 to 0.881), the criterion value (cutoff value) was 0.0386 with a sensitivity of 76.9%, and the specificity was 76.6%. The AUC of *TIMP1* was 0.767 ± 0.043 (*P* = 0.0001; 95% CI 0.682 to 0.851), the criterion value (cutoff value) was 0.215 with a sensitivity of 61.5%, and the specificity was 83.0%. The AUC of *STC2* was 0.769 ± 0.045 (*P* = 0.0001; 95% CI 0.680 to 0.858), the criterion value (cutoff value) was 0.01 with a sensitivity of 94.9%, and the specificity was 53.2%. The AUC of *SRPX2* was 0.507 ± 0.059 (*P* = 0.901; 95% CI 0.392 to 0.621). The AUC of *SPARC* was 0.452 ± 0.055 (*P* = 0.372; 95% CI 0.345 to 0.559). The AUC of *TLN2* was 0.485 ± 0.056 (*P* = 0.783; 95% CI 0.376 to 0.594).

### Expression of *KAP1*, *TIMP1* and *STC2* in patients with gastric cancer and healthy volunteers

**Figure 2:**
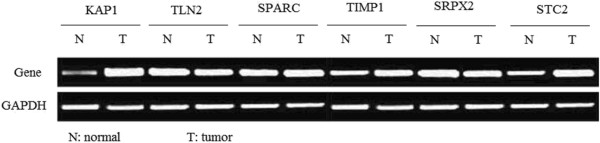
**The gel electrophoresis of *KAP1*, *TIMP1*, *STC2*, *TLN2*, *SRPX2*, and *SPARC *in patients with gastric cancer and healthy volunteers.**

According to the criterion value (cutoff value), the higher expression of *KAP1* was detected in 21 of 107 (19.6%) healthy volunteers and in 99 of 112 (88.4%) patients with gastric cancer (χ^2^ = 104.5, *P* = 0.0001) (Figure 2). The higher expression of *TIMP1* was detected in 19 of 107 (17.8%) healthy volunteers and in 100 of 112 (89.3%) patients with gastric cancer (χ^2^ = 112.8, *P* = 0.0001). The higher expression of *STC2* was detected in 20 of 107 (18.7%) healthy volunteers, and in 101 of 112 (90.2%) patients with gastric cancer (χ^2^ = 113.1, *P* = 0.0001).

### Relationship between expression of *KAP1*, *TIMP1* and *STC2* in patients with gastric cancer and clinical characteristics

We examined the correlation between the expression levels of *KAP1*, *TIMP1* and *STC2* and clinical parameters. No significant associations were found between these three mRNAs, and gender, tumor location, tumor size, Lauren classification, histology classification, differentiation or distant metastasis (*P* >0.05). There were, however, significant association between the three mRNAs and TNM stages and lymph node metastasis (Table 3).

**Table 3:** **Relationship of *KAP1*, *TIMP1 *and *STC2 *expression with pathological parameters of tumor**

**Clinical parameters**	***KAP1***				***TIMP1***				***STC2***			
	**Low**	**High**	**χ**^**2**^	***P***	**Low**	**High**	**χ**^**2**^	**P**	**Low**	**High**	**χ**^**2**^	***P***
N	13	99			12	100			11	101		
Gender			1.52	0.22			0.68	0.41			3.08	0.079
Male	7	70			7	70			5	72		
Female	6	29			5	30			6	29		
Location			0.184	0.91			1.82	0.40			1.99	0.369
Proximal	1	11			0	12			0	12		
Middle	4	32			5	31			5	31		
Distal	8	56			7	57			6	58		
Size			0.227	0.633			0.156	0.693			0.484	0.487
<5 cm	5	45			6	44			6	44		
≥5 cm	8	54			6	56			5	57		
Lauren classification			0.56	0.45			0.55	0.46			0.037	0.85
Intestinal	8	50			5	53			6	52		
Diffuse	5	49			7	47			5	49		
Histology			1.53	0.675			5.376	0.146			2.62	0.454
Papillary adenocarcinoma	0	5			0	5			0	5		
Tubular adenocarcinoma	11	69			12	68			10	70		
Mucinous adenocarcinoma	1	15			0	16			1	15		
Signet-ring cell carcinoma	1	10			0	11			0	11		
Histologic differentiation			0.013	0.91			1.97	0.16			0.10	0.75
Well-moderately	4	32			6	30			4	32		
Poorly-undifferentiated	9	67			6	70			7	69		
TNM Stages			24.31	0.001			36.46	0.001			20.26	0.001
I	7	7			8	6			6	8		
II	4	36			2	38			3	37		
III	2	37			2	37			1	38		
IV	0	19			0	19			1	18		
Lymph node metastasis			48.37	0.001			24.15	0.001			41.32	0.001
No	7	1			5	3			6	2		
Yes	6	98			7	97			5	99		
Distant metastasis			0.898	0.343			2.746	0.098			0.537	0.464
No	12	81			12	81			10	83		
Yes	1	18			0	19			1	18		

### The diagnostic efficacy of *KAP1*, *TIMP1*, *STC2*, *TLN2*, *SRPX2* and *SPARC* in patients with recurrence of gastric cancer

**Figure 3:**
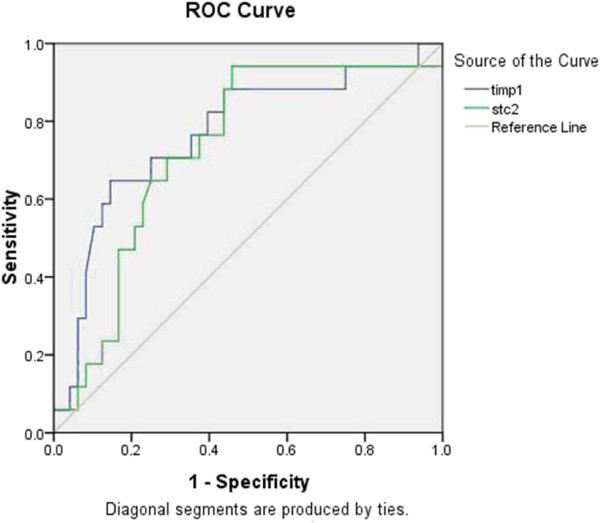
**Receiver operation characteristics (ROC) curve of the TIMP1 and STC2 in peripheral blood of patients with recurrence of gastric cancer and healthy volunteers.** The clinical values were assessed by 42 patients with recurrence of gastric cancer from 107 healthy volunteers.

To evaluate the diagnostic value of *KAP1*, *TIMP1*, *STC2*, *TLN2*, *SRPX2* and *SPARC* expression levels in the diagnosis of patients with recurrence of gastric cancer, the AUC value from ROC curve analysis was determined (Figure 3). The AUC of *TIMP1* was 0.761 ± 0.073 (*P* = 0.002; 95% CI 0.619 to 0.903), the criterion value (cutoff value) was 0.214 with a sensitivity of 64.7%, and the specificity was 83.0%. The AUC of *STC2* was 0.739 ± 0.070 (*P* = 0.004; 95% CI 0.603 to 0.875), the criterion value (cutoff value) was 0.0129 with a sensitivity of 94.1%, and the specificity was 55.3%. The AUC of *KAP1* was 0.418 ± 0.088 (*P* = 0.319; 95% CI 0.246 to 0.59). The AUC of *TLN2* was 0.441 ± 0.083 (*P* = 0.475; 95% CI 0.279 to 0.603). The AUC of *SRPX2* was 0.453 ± 0.074 (*P* = 0.569; 95% CI 0.308 to 0.599). The AUC of *SPARC* was 0.416 ± 0.080 (*P* = 0.308; 95% CI 0.26 to 0.573).

## Discussion

The outcome of patients with gastric cancer depends on tumor stage at the time of diagnosis [13]. Endoscopic examination is the most reliable method for the diagnosis of gastric cancer; however, the feasibility and cost effectiveness of this invasive approach in most countries remain questionable [14]. A simple diagnostic test, such as a serum biomarker assay, could facilitate screening for gastric cancer. Currently, however, there are no serum biomarkers that are sufficiently sensitive and specific for the routine diagnosis of gastric cancer. The sensitivities of tumor markers such as CEA, CA 19–9 and CA 72–4 are low (20 to 30%) [15-17]. O’Sullivan *et al*. [18] suggested that preoperative detection of micrometastases may reflect either transient shedding of cells, metastatic potential or residual disease.

In this study, we detected the expression levels of *KAP1*, *TIMP1*, *STC2*, *TLN2*, *SRPX2* and *SPARC* mRNAs in peripheral blood samples from pre-operative gastric cancer patients, those with recurrence, and in healthy volunteers. ROC curve analysis was used to evaluate the diagnostic value of the expression levels of these genes in differentiating preoperative gastric cancer patients or patients with recurrence from healthy volunteers. The study showed that *KAP1*, *TIMP1* and *STC2* expression levels could differentiate preoperative gastric cancer patients from healthy volunteers, and that *TIMP1* and *STC2* could differentiate preoperative gastric cancer patients with recurrence from healthy volunteers.

Previous studies showed that *TIMP1* was overexpressed in both liver and peritoneal metastases from patients with colorectal adenocarcinoma, melanoma and malignant thyroid neoplasms. *TIMP1* showed significant increase in immunoreactivity in the colorectal carcinomatous epithelium compared with the adenomatous epithelium [19]. *TIMP1* was overexpressed in both liver and peritoneal metastases from patients with colorectal adenocarcinoma [20]). The level of *TIMP1* mRNA expression was found to be an independent diagnostic marker of malignant thyroid neoplasms [21]. In our study, the expression levels of *STC2* in patients with gastric cancer were significantly higher than those in healthy volunteers. There were significant correlations between the expression levels of *STC2*, and TNM stages and lymph node metastasis. The level of serum TIMP-1 in patients with melanoma has previously been shown to be significantly higher than in controls, leading to the speculation that serum level of TIMP-1 may be a new useful marker for melanoma progression [22].

Solid tumor progression is usually associated with hypoxia. STC2 has been suggested as a novel target of oxidative stress response to protect cells from apoptosis. The expression of *STC2* has been reported to be highly correlated with human cancer development. The identification and functional analysis of *STC2* upregulation by hypoxia, a feature of the tumor microenvironment, sheds light on a possible role for STC2 in tumors. The cell proliferation was reduced in *STC2*-silenced cells but was increased in *STC2*-overexpressing hypoxic cells [23]. The stable expression of exogenous *STC2* promoted epithelial-mesenchymal transition in hypoxic conditions. When *STC2* was stably transfected into cells, they showed a high degree of motility with fibroblast morphology under hypoxic condition, and a high degree of invasiveness in hypoxia. The finding provides evidence that *STC2* is a positive regulator of tumor progression in hypoxic conditions [24]. In our study, the expression of *STC2* in patients with gastric cancer was significantly higher than in healthy volunteers. There were significant correlations between the expression of *STC2*, and TNM stages and lymph node metastasis. Previous studies have shown that the expression of *STC2* in colorectal cancer tissue was higher than in corresponding normal colorectal epithelial tissue. The high expression of *STC2* was correlated with lymph node metastasis, lymphatic invasion, tumor depth, tumor size, AJCC stage classification and worse overall survival rates [9]. *STC2* was upregulated at the mRNA and protein levels in renal cell carcinoma [25]. *STC2* was expressed in invasive breast tumor cells, while univariate survival analysis revealed that expressions of *STC2* were correlated with longer disease-free survival [26].

In our study, we focused on the *KAP1* expression patterns in peripheral blood from patients with gastric cancer. The levels of *KAP1* in the gastric cancer group were found to be significantly higher than those in the healthy volunteer group. There were significant correlations between the expression of *KAP1*, and TNM stages and distant metastasis. The study by Yokoe *et al*. showed that the expression of *KAP1* was significantly higher in cancerous tissues than in non-cancerous tissues. Patients with high *KAP1* expression showed a higher incidence of peritoneal carcinomatosis and significantly poorer overall survival compared with patients with low *KAP1* expression. Multivariate analysis revealed that high *KAP1* expression was an independent prognostic factor. Intriguingly, high *KAP1* expression was also an independent factor for peritoneal carcinomatosis [10]. To evaluate the diagnostic value of *KAP1* in the diagnosis of patients with recurrence of gastric cancer, the AUC value from ROC curve analysis was determined. The AUC of KAP1 was 0.418 ± 0.088 (*P* = 0.319), indicating that expression levels of *KAP1* could not distinguish recurrence of gastric cancer from cancer, probably because the number of recurrences of gastric cancer were too few. The proliferation rate was impaired and resistance to anoikis was decreased after knockdown of *KAP1* in the gastric cancer cell lines AZ521 and KATO III [10]. *KAP1* contributes to the negative regulation of E2F1 and may serve as a partial backup to prevent E2F1-mediated apoptosis in the absence of pRb [27].

Previous studies have shown that *SRPX2* and *SPARC* were overexpressed in gastric cancer tissues [28-30], while *TLN2* was upregulated in breast carcinomas tissues [31]; however, no studies investigating the expression of *TLN2*, *SRPX2*, and *SPARC* in peripheral blood have been reported. Our study showed that *TLN2*, *SRPX2* and *SPARC* expression were detected in gastric cancer, but expression of *TLN2*, *SRPX2* and *SPARC* could not differentiate preoperative gastric cancer patients from healthy volunteers.

*KAP1*, *TIMP1* and *STC2* appear to have a role in the progression to metastatic disease in gastric cancer. Preoperative *KAP1*, *TIMP1* and *STC2* expression levels in peripheral blood were related to cancer stage and may be markers of tumor invasion, lymph node metastasis and TNM stage. In particular, high *TIMP1* and *STC2* expression levels could be poor prognostic factors of disease recurrence in patients with gastric cancer.

## Conclusions

Expression levels of *KAP1*, *TIMP1* and *STC2* are potentially useful as clinical biomarkers for the screening, diagnosis, prognostic and surveillance of gastric cancer.

## Abbreviations

AJCC: American Joint Committee on Cancer; AUC: Area under the ROC curves; bp: Base pairs; EMT: Epithelial-mesenchymal transition; ITGB1: Integrin beta 1; KAP1: Kinesin II-associated protein; MMPs: Matrix metalloproteinases; ROC: Receiver operation characteristics; SPARC: Secreted protein, acidic, cysteine-rich; SRPX2: Sushi-repeat-containing protein, X-linked 2; STC2: Stanniocalcin 2; TIMP: Tissue inhibitor of metalloproteases; TIMP1: TIMP metallopeptidase inhibitor 1; TLN2: Talin 2

## Competing interests

We declare that we have no conflicts of interest.

## Authors’ contributions

Y-YW, LL, and H-JW participated in the scientific experiments. All authors participated in the conception and design of the study, as well as data collection and interpretation, manuscript preparation and literature search. All authors have read and approved the final manuscript.
